# Comparison of Zirconia Implant Surface Modifications for Optimal Osseointegration

**DOI:** 10.3390/jfb15040091

**Published:** 2024-04-02

**Authors:** Hyun Woo Jin, Sammy Noumbissi, Thomas G. Wiedemann

**Affiliations:** 1 BA/DDS Joint Program, College of Dentistry, New York University, New York, NY 10010, USA; 2 Department of Oral and Maxillofacial Implantology, University of Milan, 20122 Milano, Italy; 3 Post-Graduate Program Oral Surgery, University of Chieti-Pescara, 65127 Pescara, Italy; 4 Department of Biomaterials INSA Lyon, 69100 Villeurbanne, France; 5 Private Practice, Silver Spring, MD 20910, USA; 6 Department of Oral and Maxillofacial Surgery, College of Dentistry, New York University, New York, NY 10010, USA

**Keywords:** zirconia implant, ceramic implant, dental implant, implant surface, implant design, osseointegration, surface modification, surface design, biological response

## Abstract

Zirconia ceramic implants are commercially available from a rapidly growing number of manufacturers. Macroscopic and microscopic surface design and characteristics are considered to be key determining factors in the success of the osseointegration process. It is, therefore, crucial to assess which surface modification promotes the most favorable biological response. The purpose of this study was to conduct a comparison of modern surface modifications that are featured in the most common commercially available zirconia ceramic implant systems. A review of the currently available literature on zirconia implant surface topography and the associated bio-physical factors was conducted, with a focus on the osseointegration of zirconia surfaces. After a review of the selected articles for this study, commercially available zirconia implant surfaces were all modified using subtractive protocols. Commercially available ceramic implant surfaces were modified or enhanced using sandblasting, acid etching, laser etching, or combinations of the aforementioned. From our literature review, laser-modified surfaces emerged as the ones with the highest surface roughness and bone–implant contact (BIC). It was also found that surface roughness could be controlled to achieve optimal roughness by modifying the laser output power during manufacturing. Furthermore, laser surface modification induced a very low amount of preload microcracks in the zirconia. Osteopontin (OPN), an early–late osteogenic differentiation marker, was significantly upregulated in laser-treated surfaces. Moreover, surface wettability was highest in laser-treated surfaces, indicating favorable hydrophilicity and thus promoting early bone forming, cell adhesion, and subsequent maturation. Sandblasting followed by laser modification and sandblasting followed by acid etching and post-milling heat treatment (SE-H) surfaces featured comparable results, with favorable biological responses around zirconia implants.

## 1. Introduction

Titanium remains the implant material of choice in dental implantology since its first use in 1965 [1]. However, zirconia development and use as a load-bearing ceramic implant started in the mid-1960s [2,3], and it has evolved rapidly during the last twenty years since its first use in 1987 [4]. Zirconia implants not only present clinical success and functional resilience—high flexural and compressive strength—but also have high esthetic value [5,6]. Zirconia implants also exhibit superior biocompatibility and soft tissue response thanks to keratinized and vascularized features [6,7]. Moreover, with superior soft tissue emergence and the absence of grey shadows as typically seen around titanium implants, ceramic implants significantly elevate the overall esthetic standards. Patients allergic or intolerant to titanium and their alloys now have an alternative material for implant-supported restorations. As discussed in Wiedemann et al. [6,8,9], implanted metals are known to undergo a slow release of ions, an issue that is also observed in titanium implants [9,10]. In fact, titanium particles and ions released from an implant can not only induce systemic allergic reactions but also trigger an inflammatory cascade that results in bone resorption as a result of elevated osteoclastic activity [8]. As such, zirconia implants in terms of aesthetics and biocompatibility stand in a better position than titanium implants.

There are three levels of implant surface characteristics: macroscopic, microscopic, and nanoscopic. Macroscopic features mainly pertain to thread design; micro and nanoscopic features relate more to the implant surface topography after etching or sandblasting protocols. Besides implant macro-design, surface topography is a key element in achieving biological and functional osseointegration—the direct structural and functional connection between ordered, living bone and the surface of a load-carrying implant [11]. Similar to their titanium counterparts, treated zirconia implants exhibit a higher BIC value than machined surfaces [12]. Unlike metal implants, the surfaces of commercially available ceramic implants are enhanced using subtractive protocols. With continued development and an increasing number of commercially available zirconia implant systems, it is crucial to evaluate and compare different types of surface modifications with regard to successfully achieving and maintaining osseointegration.

Bone–implant contact (BIC) is widely used to describe the degree of osseointegration. The aim of this article is to compare the BIC value between different implant systems in zirconia micro-surface designs (i.e., surface roughness, hydrophilicity) and the associated biological responses due to different study designs and difficulties in comparing and fully differentiating zirconia surface designs.

It is widely accepted that the degree of osseointegration is largely dictated by surface roughness, energy, hydrophilicity, and biological response [13,14]. In fact, as discussed in Jing et al., a micro–nano scale surface design is an effective method to fabricate surfaces to promote an implant’s performance in terms of wettability and frictional enhancement [15]. There is a general consensus that rougher surfaces provide improved osseointegration characteristics with higher bone anchorage potential. However, research indicates that excessive roughness achieved by extensive surface treatment may result in micro-cracks and defects, leading to the deterioration of the mechanical properties [16]. Monoclinic phase (*t-m*) transformation reflects zirconia’s susceptibility to fracture. A *t-m* transformation is a property change from a tetragonal crystal phase (t) to a monoclinic crystal phase (m) [17]. Such transformation in low-temperature degradation (LTD), which takes place by hydrothermal aging in a moisture-excess environment like the oral cavity, is known to be determinantal to mechanical stability [18]. As such, it is important to obtain optimal surface roughness, which is described as a “moderately roughened surface” by Wennerberg et al. [19] Optimal surface roughness (S_a_—arithmetic mean height deviation from mean plane value) for zirconia implants was found to be at a range of 1–1.5 μm, showing that 1.5 μm of roughness exhibited the most favorable bone in-growth [20].

Hydrophilicity, as discussed in Moura et al., has an intimate relationship with enhancing the surface energy of implants and thus improving osteogenic cell adhesion [21]. Hydrophilicity is measured by the degree of surface wettability. Interestingly, surface wettability has a direct relationship with surface roughness [22]. Surface wettability is typically measured by the water contact angle with a device using AutoCAD 2010 software [21]. A contact angle above 90° is considered “hydrophobic”, while an angle below 90° is considered “hydrophilic”. In studies conducted by Zhao et al. (2005) and Wu et al. (2015), hydrophilic surfaces resulted in a higher differentiation of the osteoblast cell phenotype, an increased degree and rate of bone formation, and improved overall osteogenic cell attachment [23,24].

The objective of this study was to compare modern surface modifications that are featured in commercially available ceramic implant systems and combinations of surface treatments with regard to favorable biological responses and osseointegration outcomes.

## 2. Materials and Methods

**Figure 1:**
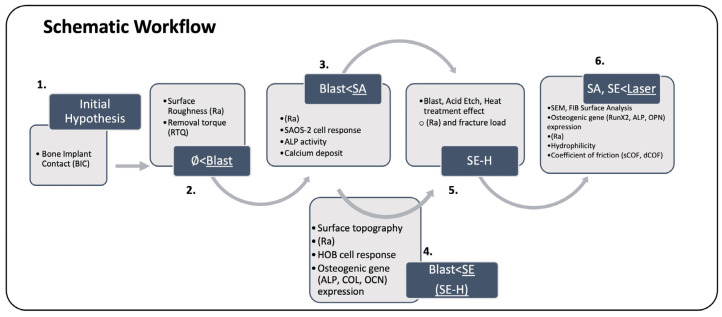
Schematic workflow.

An electronic search was conducted using search engines such as PubMed, Cochrane, Google Scholar, Elsevier’s platform, and Wiley Library. Keywords included “Ceramic” OR “Zirconia” AND “Implant” AND “Surface” AND “Design” AND “Modifications”. Inclusion criteria were a publication year ranging between 2012 and 2023. Only systematic reviews, meta-analyses, clinical trials, and in vitro and in vivo studies with at least a 6-week follow-up for bone–implant contact (BIC) and removal torque measurements (RTQ) presented in the English language were considered. Exclusion criteria included a less than 4-week follow-up for BIC and RTQ assessment and information pertaining to titanium-based implants. Sixteen articles of interest were selected after assessing the abstracts and subsequently reading the full texts to determine their relevance to this study. To ensure that relevant articles were not missed, studies indicated in the reference list were reviewed as well. Moreover, articles that communicated associated biological factors, physical properties, and surface topographies were further hand-searched and selected without date restriction. The obtained information was evaluated for its significance and relevance to this study. This study was conducted as illustrated in the schematic workflow in Figure 1. The comparison of zirconia implant surface designs was made considering bio-mechanical factors such as null (machined) surface, blasted surface, acid etch (SA) surface, blast followed by acid etch (SE) surface, SE followed by heat treatment (SE-H) surface, and laser-treated surface.

Data were extracted from the selected articles. Data categorization was conducted by sorting implants according to surface characteristics. BIC values were compared to obtain a general perception of the degree of osseointegration for each type of surface modification method used. As this study’s interest lay in conducting a detailed comparison of surface modifications, bio-physical properties (surface roughness, design, and hydrophilicity) were evaluated when making this comparison.

## 3. Results

In total, five distinct surface modifications presented in five selected commercially available zirconia implant systems were evaluated (Table 1).

A blasted surface design was featured for a variety of manufacturers: the SA surface by CeraRoot SL^TM^, SE design by Nobel Biocare Nobel Pearl^TM^, and Dentalpoint AG’s Zeramex XT^TM^. It was noted that Zeramex and Nobel Biocare, which are distinct companies, utilized the same foundational micro implant design supplied by Dentalpoint AG^TM^. SE-H modification was used by Straumann Pure^TM^, while a laser-ablated design was applied by Straumann Snow^TM^.

The following were appraised for clinical applicability, in terms of success of osseointegration: 1. blast, 2. acid etch (SA), 3. blast followed by acid etch (SE), 4. SE followed by heat treatment (SE-H), and 5. blast followed by laser irradiation.

### 3.1. Comparison of Bone–Implant Contact

An overall assessment of osseointegration potentiality was performed by comparing BIC values, as seen in Table 2.

There were no articles found in the literature that compared all surface modifications in zirconia implants under the same testing conditions. Thus, only articles with a BIC comparison using the same testing environment were selected. Despite all efforts, a comparison of BIC in “blasted vs. SA”, “SA vs. SE”, and “SE vs. Laser treated” surfaces could not be identified. Therefore, a preliminary hypothesis was established that the most favorable outcome would be featured in a laser-ablated > SE-H > SE > SA > blasted > null (machined) surface. The following sections will compare the major bio-physical properties presented in a number of studies to closely evaluate these surface modifications.

### 3.2. Comparison of Machined and Blasted Surfaces

As presented by Gahlert et al. [27], surface roughness and removal torque were assessed for machined and Al_2_O_3_ (250 μm diameter, 5 bar) blasted zirconia implants.

The values of surface roughness (Ra) were 0.13 μm in machined and 0.56 μm in blasted zirconia dioxide implant surfaces [27].

The mean removal torque (RTQ), calculated through 3 follow-ups (4, 8, and 12 weeks), was higher for a blasted surface (40.5 N/cm) than for a machined surface (25.9 N/cm) [27].

In addition, when BIC values were visually compared with cross-sectional histological analysis, blasted surfaces featured higher BIC degrees in the 12-week follow-up.

### 3.3. Comparison of Blasted and SA Surfaces

Hempel et al. compared the surface characteristics of blasted and SA surfaces. Surface irregularity and roughness were visually and arithmetically examined. Through SEM imaging, the authors reported that blasted surfaces showed a higher level of irregularity and a coarse structure compared to etched surfaces. With these visual findings, they found that surface roughness was higher for blasted (Ra = 1.13 μm) compared to Sa (Ra = 1.11 μm) surfaces [28].

Furthermore, the degree of cell adhesion and proliferation was assessed through fluorescence imaging. Both surfaces exhibited well-organized actin fibers of SAOS-2 cells within 2 h after seeding. In 24 h follow-ups, both surfaces exhibited SAOS-2 cell spreading, with numerous focal adhesion contacts [28]. The differences in the cell proliferation rate were assessed after 2, 4, and 24 h. Although the same proliferation rate was observed on both surfaces in the 2 h and 24 h follow-ups, a higher rate was observed on SA surfaces in the 4 h follow-up. The proliferation rate of SAOS-2 cells was measured to be higher on an SA surface (9739 ± 217 c.p.m/24 h) compared to a blasted surface (9107 ± 351 c.p.m/24 h) [28].

Finally, the osteogenic ALP activity and calcium deposit rate were evaluated. SAOS-2 cell ALP activity was higher for SA surfaces compared to blasted surfaces.

Cell-associated calcium deposits were higher for an SA surface (4.62 ± 0.13 μmol/sample) than for a blasted surface (4.45 ± 0.12 μmol/sample) [28].

### 3.4. Comparison of Blasted, SE, and SE-H Surfaces

Bergemann et al. [29] compared four zirconia implant surfaces: machined, blasted, SE, and SE-H. The comparison was performed by examining surface topography, HOB cell response, and osteogenic gene activity. Through SEM imaging and confocal laser microscopy, surface geography was assessed visually and numerically.

Surface roughness was highest in SE-H > SE > blasted > machined surfaces (Table 3). More porous structures were featured in SE and SE-H surfaces. In fact, the additional acid etch treatment conducted for SE and SE-H not only increased the surface roughness but also smoothened the sharp edges that were observed on blasted surfaces [29].

The HOB cell response was assessed by cell spreading, cell anchorage, and actin filament length. HOB cell spreading was highest in machined surfaces compared to other designs. Additional heat treatment as performed for SE-H showed marginal improvement in cell spreading. This was explained by a higher degree of cell anchorage with increased surface roughness. Consistent with the cell spreading and cell anchorage outcome, a shorter actin stress filament was observed on a rougher surface. As presented in Table 3, the average actin filament length was longest in machined > blasted > SE-H > SE surfaces in 24 h follow-ups [29].

Noting the “reversible effect” featured in heat treatment, surface irregularity was determined by the orientation dispersion value. Surface irregularity was highest in SE > blasted > SE-H > machined surfaces (Table 3) [29]. Interestingly, additional heat treatment as performed for SE-H surfaces not only improved surface roughness but also restored the unfavorable effect in HOB cell spreading, actin filament length, and surface irregularity.

Finally, osteogenic differentiation marker (ALP, COL, and OCN) activities were assessed throughout a 3-day period. Early differentiation markers, such as ALP- and COL activity, were significantly down-regulated in SE and SE-H surfaces, while a late differentiation marker, OCN activity, was significantly up-regulated in SE and SE-H surfaces [29].

### 3.5. Evaluation of Blast, Etch, and Heat Treatment

The above studies demonstrated that SE and SE-H surfaces feature promising results compared to blast-treated surfaces. However, additional information on the degree of surface treatment is necessary in making a comprehensive comparison. Fischer et al. [30] conducted a study determining the effect of sandblasting with subsequent acid etching and heat treatment on bio-physical properties.

#### 3.5.1. Blast Treatment

Increasing the number of blast cycles improved the surface roughness up to 1.2 μm, which was the maximum attainable roughness. Further increases in roughness were not observed after the fourth cycle. Monoclinic transformation continued to increase with the number of blast cycles. Fracture load continued to increase up to the sixth cycle [30]. In summary, four blast cycles were deemed ideal blast treatment for zirconia surfaces.

#### 3.5.2. Acid Treatment

The effects of an additional HF acid etch treatment were assessed in obtaining an SE surface. Blast surfaces manufactured with four and twenty cycles were selected for evaluation. No significant change was observed in surface roughness with etching time. Monoclinic fraction increased in blast surfaces with four cycles and decreased in surfaces with twenty cycles after 1 h of etching time. No further change in monoclinic transformation was achieved after 1 h. Fracture load continued to decrease in both subjects with continued exposure to etching treatment [30].

#### 3.5.3. Heat Treatment

For the obtained SE surfaces (4 × blast and 1 h etching surface and 20 × blast and 1 h etching surface), an additional heat treatment was implemented. There was no significant change in microstructure surfaces with annealing. A significant reduction in monoclinic fractions was observed at 1 h for both subjects: from 14.5% to 2.4% for 4-cycled SE-H surfaces and from 14.7% to 1.6% for 20-cycled SE-H surfaces. Heat treatment beyond 1 h re-increased the monoclinic fraction. Fracture load decreased in both surfaces with 1 h of annealing treatment. No further change in fracture load was observed after a 1 h treatment [30].

In summary, 4-cycle-blasted, 1 h HF-etched, and 1 h heat-treated SE-H showed the most promising results, with favorable surface roughness, monoclinic fractions, and fracture load. This supports the results found in previous studies, in which an SE-H was shown to have more favorable bio-mechanical properties compared to an SE surface.

### 3.6. Comparison of SA, SE, and Laser-Treated Surfaces

Monzavi et al. [31] assessed four types of zirconia implants, as indicated in Table 4.

These four different types of implant systems and microstructures were compared by SEM analysis and FIB analysis. The change in microstructural properties was assessed after accelerated aging tests. Accelerated aging was featured by an environment of water steam at 134 °C under a 2-bar pressure: 1 h of such conditions reflected approximately two years of aging under a body temperature of 37 °C; 15 and 30 h of accelerated aging, which reflected approximately 30 and 60 years, respectively, were implemented as well [31].

Through SEM analysis, on a laser-modified surface (type B), notably fewer micro cracks and a microporous surface structure were observed. A significant finding was noted in an SE (type D) modification exhibiting a relatively smoother surface compared to other implant surface types, featuring a regular pattern of surface roughness with shallow grooves with fewer micro cracks [31].

With FIB analysis, the degree of micropores and microcracks and the average grain size were assessed in selected implants as shown in Table 5. A laser-finished surface (type B) featured the smallest average grain size of 0.25 μm. Interestingly, the SE finish (type D) exhibited no micropores, but the microcrack depth averaged 0.7 μm and was the highest [31].

FIB analysis of cross-sections of surfaces of aged implants was conducted as in Table 6.

At 15 h, the laser-finished surface (type B) featured a micro crack depth of 0.7 μm. At 30 h, microcracks were relatively consistent at the 15 h mark, with average values of 0.6 μm [31]. Laser-treated surfaces resulted in distinctive features, with relatively consistent microcracks; in fact, they featured lower *t-m* transformation after accelerated aging.

Noting the favorable surface topography of laser-finished surfaces, an evaluation of osteogenic potential was deemed necessary. A study by Sun et al. compared polished, blasted, SE, and laser-modified surfaces. MC3T3-E1, mouse osteoblast-like cells, were utilized to assess cell response and the level of osteoblastic differentiation. After a 7-day follow-up, cell proliferation was the highest in laser-treated > SE > blasted > polished surfaces [32]. For the evaluation of osteogenic potential, the activities of three types of osteogenic differentiation genes—runt-related transcription factor 2 (RunX2), alkaline phosphatase (ALP), and osteopontin (OPN)—were compared. All three osteogenic gene expressions were highest in laser-treated > SE > blasted > polished surfaces [32].

Many studies accord that a laser ablation technique can be meticulously controlled by altering the irradiation intensity. A study conducted by Moura et al. included two laser-treated surfaces designed under different laser irradiation power outputs, L2 (1.8 W) and L1 (0.9 W). The authors compared four types of zirconia implant surfaces: SE and machined surfaces in terms of surface topography, surface wettability, monoclinic content, and coefficient of friction. As presented in Table 7, the highest surface roughness was seen in laser-irradiated L2 > SE > laser-irradiated L1 > blasted surfaces. There was no significant change in post-aged surfaces [21].

For hydrophilicity or surface wettability, the water contact angle was examined. The lowest water contact angle was observed in L2 < L1 < SE < machined surfaces. Favorable hydrophilicity was observed in laser-treated surfaces.

Finally, to evaluate the degree of osseointegration, the dynamic and static coefficients of friction, dCOF and sCOF, respectively, were assessed. dCOF was highest in L2 (0.4) > SE > L1 > machined surfaces. sCOF was highest in L2 (0.66) > SE > L1 > machined surfaces [21].

## 4. Discussion

The objective of this study was to conduct a comparison of five types of surface modifications currently available for modern zirconia implant systems. Initially, osseointegration capacity was compared via the BIC value in order to construct a preliminary hypothesis. As zirconia is a more contemporary implant material, the currently available literature lacks studies that compare the BIC value for a variety of surface modifications under the same testing conditions. Thus, data were obtained from different studies that compared at least two surface designs under the same testing conditions. Comparable BIC data for blasted vs. SA surfaces, SA vs. SE surfaces, and SE vs. laser-treated surfaces could not be acquired. Using only BIC values, it was hypothesized that the osseointegration capability is highest in laser-modified > SE-H > SE > blasted ~ SA > machined surfaces [25,26].

During the osseointegration process, primary and secondary stability are relevant to implant success. It is widely accepted that favorable primary stability, which is highly dictated by mechanical engagement during the implantation procedure, affects secondary stability, which is achieved through the biological response after implant surgery [33]. When an implant is placed, its primary stability is derived mainly from its macroscopic features such as threads, taper, and shape. After a few weeks post-placement, the biological stability process begins to take effect, with bone attaching to the implant surface. BIC histological analysis is mainly used to evaluate how much of an implant surface has been “colonized” by bone. Therefore, it can be concluded that the degree of implant stability observed in osseointegrated implants is a clinical manifestation of optimal BIC and osseointegration. However, as discussed by Swami et al. [34], BIC histological analysis, despite its accurate measurement, is invasive and destructive in nature and is not suitable for long-term evaluation [34]. Thus, other factors that include mechanical configuration and biological response are more critical to comprehensively extrapolate the degree of osseointegration.

Surface roughness is one of the most critical factors in the micro-design of implants. It is widely accepted that the increase in surface roughness accelerates osseointegration [12]. In fact, as discussed in Wennerberg et al. [20], approximately 1.5 μm of surface roughness favors bone growth the most [20]. The surface roughness of SE, SE-H, and laser-treated surfaces showed optimal values for osseointegration [21,29]. The different levels of surface roughness obtained for laser-treated surfaces L1 and L2 [21] suggest that optimal surface roughness can be achieved by controlling the power output.

As discussed in Wennerberg et al., it is important to acknowledge that an optimal surface is a “moderately roughened surface” [20]. In fact, a study by Monzavi et al. [31] further discusses that while surface roughness may improve an implant’s osseointegration potential, susceptibility to water penetration, and aging, LTD may also increase [31]. Although accelerated aging tests (Table 5 and Table 6) affected the zirconia implant grain transformation, the effect was minimal. Thus, it was proposed that resistance to aging or LTD may be more related to structural composition than surface topography [31].

Osteogenesis is one of the primary factors in osseointegration, with new bone formation besides the synthesis and deposition of an extracellular matrix [35]. As discussed by Huang et al. [35], sequential osteoblastogenesis of proliferation, matrix maturation, and mineralization should be appreciated. Common osteogenic differentiation markers include RunX2 as an osteoblast differentiation inducer, ALP and COL for the early phase, OCN for the late phase that coincides with mineralization, and OPN for both early and late stages [35]. As discussed by Bergemann et al. [29], SE and SE-H surfaces initially exhibited high ALP and COL levels (subsequently down-regulated) and high OCN levels in the late stage (up-regulated) [29]. In a study by Sun et al. [32], laser-treated surfaces showed more favorable levels for all three differentiation markers (RunX2, ALP, and OPN), suggesting that osteogenic capacity is most favorable in laser-treated surfaces [32].

A study by Bergemann et al. [29] suggests that additional heat treatment for SE-H surfaces results in a favorable so-called “reversible effect” by improving surface properties and cell spreading comparable to an SE surface. In a study by Moura et al. [21], favorable hydrophilicity was observed in laser-treated surfaces, which suggests appropriate surface energy for osteogenic cell adhesion.

The osseointegration capacity of zirconia implants is determined by multiple factors [36]. Although the surface design is one of the most relevant elements affecting osseointegration, other constituents such as the thread design, implant length, and implant diameter play key roles in maximizing the functional surface area for bone integration and therefore affect bone–implant contact and biological response as well [36]. Therefore, it is essential to encompass all aspects of zirconia implant designs to fully compare and appreciate implant surfaces in regard to osseointegration.

As already performed for SE-H surfaces in a study by Fischer et al. [30], future studies should focus on comparing the extent of blast and power output for laser-treated surfaces and subsequently establish an appropriate manufacturing sequence that promotes the most favorable bio-mechanical outcomes. Since SE-H and laser-treated surfaces are already presenting highly promising results, it would be very appropriate to compare these two surface designs in regard to BIC and bio-physical components in the same testing environment.

## 5. Conclusions

All investigated surface modifications showed improvement in osseointegration potential when compared to a machined surface design. Superior outcomes were achieved in laser-treated> SE-H > SE > SA ~ blasted surfaces. The assessment of the surface topography, biological response, hydrophilicity, and coefficient of friction favored a laser-treated zirconia implant surface design. A laser modification technique has significant advantages in achieving a micro-sensitive surface design and long-term stability.

Despite efforts, relevant studies that compare blasted vs. SA surfaces and SE-H vs. laser-treated surfaces were not identified. The SE-H surface seems to be advantageous since heat treatment not only improved the surface topography but also reversed the unfavorable outcome that was observed in SE-only-treated surfaces.

Future studies that compare all proposed surface designs under the same test conditions with BIC including bio-mechanical factors would further deepen our knowledge of surface modifications in zirconia implants. Moreover, establishing a preparation sequence for laser treatment would allow for optimal manufacturing instructions in achieving favorable biological responses.

According to the currently available literature, a zirconia implant surface achieved with “blast and laser irradiation” and “blast followed by acid etch and heat treatment (SE-H)” showed the best prospective outcomes for clinical applications.

## Figures and Tables

**Table 1:** Selected implant systems and surface properties.

Manufacturer	Brand Name	Material	Surface Design
Straumann	Pure^TM^	HIP TZP	Sandblasting + Heated Acid Etching (ZLA)
	Snow^TM^	HIP TZP	Sandblasting + Laser Irradiation
Dentalpoint AG	Zeramex XT^TM^	ATZ	Sandblasting + Acid Etching (Zerafil)
Noble Biocare	Noble Pearl^TM^ (Dentalpoint AG)	ATZ	Sandblasting + Acid Etching (Zerafil)
CeraRoot SL	CeraRoot^TM^	3Y-HA	Acid Etching (ICE Surface)

**Table 2:** BIC (in %) values in different types of zirconia surface modifications [25,26].

**Machined**		**SA**	
62.14 ± 2.8%		75.01 ± 5.1%	
6 wk			
**Blast**		**SE**	
54.6 ± 17.6%		57.6 ± 23.7%	
13 wk, SA (1.0 μm; 1.2 μm)			
**Machined**	**Blast**		**Laser-modified**
32.996 ± 14.192%	39.614 ± 15.029%		39.965 ± 13.194%
Z Systems AG, 6 wk			

**Table 3:** Surface roughness (Ra), mean HOB cell actin filament length, and orientation dispersion value in machined, blasted, SE, and SE-H surfaces [29].

	Machined	Blasted	SE	SE-H
R_a_	0.59 μm	1.22 μm	1.31 μm	1.32 μm
Mean Filament Length	36.6 ± 9.8 μm	32.9 ± 5.2 μm	22.1 ± 6.8 μm	29.4 ± 3.8 μm
Orientation Dispersion Value	14.8 ± 3.8°	19.3 ± 3.9°	21.6 ± 1.8°	17.7 ± 4.9°

**Table 4:** Four types of zirconia implants (Type A, B, C, and D) [31].

	System	Material and Surface Treatment
Type A	Z systems with HIP TZP-A	Laser-Treated
Type B	Straumann Pure^TM^ with TSZ	Sandblasted + Laser-Treated
Type C	Ceraroot^TM^ with TSZ	Acid Etched Patented ICE Surface^®^
Type D	Zeramex Dental Point AG^TM^ with TSZ	Al_2_O_3_ Sandblasted + H_3_PO_2_ Acid Etched

**Table 5:** Microcrack depth and grain size in different zirconia surface designs [31].

	Type A	Type B	Type C	Type D
**Microcrack**	0.5 μm	0.5 μm	0.3 μm	0.7 μm
**Grain size**	0.35 μm	0.25 μm	0.35 μm	0.35 μm

**Table 6:** Microcrack depth after accelerated aging (15 h/30 h) [31].

	Type A		Type B		Type C		Type D	
**Aging Time**	15 h	30 h	15 h	30 h	15 h	30 h	15 h	30 h
**Microcrack**	0.9 μm	1 μm	0.7 μm	0.6 μm	0.3 μm	1.5 μm	0.8 μm	1.3 μm

**Table 7:** Surface roughness (initial and post-accelerated aging) [21].

**Initial**	**Machined**	**SE**	**L1**	**L2**
R_a_	0.43 ± 0.10 μm	1.24 ± 0.18 μm	1.06 ± 0.20 μm	2.66 ± 0.31 μm
**Aging**	**Machined**	**SE**	**L1**	**L2**
R_a_	0.39 ± 0.25 μm	1.38 ± 0.23 μm	1.04 ± 0.19 μm	2.82 ± 0.38 μm

## Data Availability

The original contributions presented in the study are included in the article, further inquiries can be directed to the corresponding author.
